# Design and Development of a Tri-Axial Turning Dynamometer Utilizing Cross-Beam Type Force Transducer for Fine-Turning Cutting Force Measurement

**DOI:** 10.3390/s22228751

**Published:** 2022-11-12

**Authors:** Muhammad Rizal, Jaharah A. Ghani, Amir Zaki Mubarak

**Affiliations:** 1 Department of Mechanical Engineering, Faculty of Engineering, Syiah Kuala University (USK), Darussalam, Banda Aceh 23111, Indonesia; 2 Department of Mechanical and Manufacturing Engineering, Faculty of Engineering and Built Environment, Universiti Kebangsaan Malaysia, UKM, Bangi 43600, Malaysia

**Keywords:** tri-axial turning dynamometer, piezoresistive strain gauge, cross-beam force transducer, fine turning process, cutting force measurement

## Abstract

The main focus of this work was the design and development of a cross-beam force transducer for use in the construction of a tri-axial dynamometer. This dynamometer would be able to measure the cutting force along all three axes simultaneously during turning operations. The force transducer was built on the concept of the Maltese cross-beam, but it had been modified and improved so that it had a higher sensitivity and reduced the amount of interference error or cross-talk error that it produced. An investigation into the distribution of strain, as well as the determination of sensor locations within the transducer construction was carried out by means of finite element analysis. In order to develop a prototype of a turning dynamometer, a number of piezoresistive strain gauges were utilized in the transducer. In order to determine sensitivity, linearity, hysteresis, and repeatability, calibration tests were performed in three directions that were perpendicular to one another. To investigate the dynamic properties and capabilities of the dynamometer for use in turning applications, both modal analysis and actual turning tests were performed. The results of the experiments demonstrated that the newly developed turning dynamometer is a realistic approach for measuring cutting force in machining without reliability and accuracy.

## 1. Introduction

In recent years, the manufacturing industry all over the world has gotten significantly more competitive, making it very necessary for enterprises operating in this industry to increase their quality and productivity while simultaneously reducing their overall production costs. Moreover, as the machining system becomes more complex and automated, there is a growing need for an in-process condition monitoring system that can provide precise and accurate speed data. This would make it so that there is no need for humans to interact with the production process at all, which would help achieve the goal of fully automating it.

The cutting force is a physical parameter that is very important during machining and becomes an excellent indicator of the overall state of the machining process, including the cutting tool condition, the quality of the workpiece, and the machine tool’s energy efficiency [1]. By implementing an accurate and reliable cutting force measuring system, the state of the cutting process can be monitored and the quality of the machined product can be ensured. In response to this, several researchers in both academic and industrial settings have suggested a variety of approaches for measuring the cutting force in a turning process. 

The cutting force measuring method for the turning process employing piezoelectric crystal materials was developed and commercialized by the industry, and it was also studied by a number of earlier researchers. Totis et al. [2] proposed a dynamometer using a triaxial piezoelectric ring placed in a modular cartridge containing an intermediary element that had been installed in a rotating turret. They reported that the natural frequency of the structure was in the range of about 544–984 Hz. Horváth et al. [3] developed a dynamometer for fine cutting force measurements. A piezoelectric cell was inserted beneath the cutting insert’s modified tool holder design. The cutting force was determined by the magnitude of the electrical charge produced by the piezoelectric crystal upon mechanical deformation. Chen et al. [4] also developed a cutting force measurement system for micro cutting by utilizing three piezoelectric ceramics. Gong et al. [5] inserted a piezoresistive ceramic-based SiAlCO directly beneath the insert in order to measure the cutting force in turning; nevertheless, its detectable range was limited to around 10 N. Although the piezoelectric crystal has good dynamic measurement sensitivity, it has limitations, including weak charge unidirectionality, which led to a significant interference problem when the force signal was recorded in three directions [6]. PZT piezoelectric film has also been used in attempts to measure the cutting force by placing it under the cutting tool insert [7]. Chen et al. [8] also utilized two piezoelectric films to detect cutting and feed forces in micro turning. However, the piezo-film sensor is only capable of measuring very low cutting forces and, as a result, the structure’s stiffness was diminished. 

Recent years have seen an increase in the utilization of a variety of thin-film strain sensors as a result of the development of microelectromechanical systems (MEMS) technology. Li et al. [9] embedded a nickel–chromium thin-film sensor on a steel substrate and screwed it on a modified tool shank with holes and cavities. Zhang et al. [10] also proposed a cutting force measuring system using eight Ni–Cr-based strain film sensors integrated into a turning tool shank. The sensors were attached to the typical octagonal link plates that were located on both ends of the elastomeric material. Cheng et al. [11] presented three types of embedded strain sensor models using a thin layer of Ni–Cr alloy. They examined the insert type, the quick insert type, and the elastic sleeve type before optimizing six shapes of the substrate structure. They found that substrate structural characteristics were the primary factors influencing the performance of thin-film strain sensors. However, they did not represent performance very well in terms of three-axis cutting force measurements. Moreover, when certain grooves and holes were added to the conventional tool holder, the dynamic performance had to be examined further.

In prior work, three-axis cutting force measurement was established by employing several elastic element designs as transducers and strain sensors. According to Yaldiz and Ünsacar [12], the approach technique makes use of an octagonal ring transducer. In this method, strain gauges are set on four octagonal rings, which serve as the transducers, and are accompanied by two plates. Rizal et al. [13] also designed and tested a three-axis strain gauge-based dynamometer for measuring the cutting force in a turning operation by employing four octagonal rings with a tool shank mounted on the front plate. The concept of an octagonal ring structure was taken by Zhao et al. [14] and modified into two mutually perpendicular octagonal rings. These rings each include a slot that is equipped with two tapping holes for the purpose of preserving the cutting tool shank. Based on the performance shown by the type of transducers using an octagonal ring shape, it was found that the construction of this dynamometer had a frequency response that was quite adequate for the turning process, which was around 765–1100 Hz. Notwithstanding this, the force measurement had relatively low sensitivity, and the interference error between channels was still quite high. 

To overcome the low sensitivity that results from the use of a metal foil strain gauge, an MEMS-based semi-conductive strain sensor was chosen and attached to an elastic element made up of two mutually perpendicular octagonal rings [15,16]. According to the findings, the sensitivity of the sensor was around 27–30 times higher than sensors that have been introduced in the past. However, the cross-interference error that occurs is extremely high. Because the octagonal ring does not have a compliant structure, any deformation in one direction will have an effect on the surface in the other direction. Significant aspects in the design of an elastic element or force transducer include the geometry and shapes of the transducer structure, the type of sensor used, since they will affect sensitivity, interference of channels, stiffness of the structure, and its ability to support dynamic loads.

In this study, a novel three-axis cross-beam force transducer based on a piezoresistive strain gauge has been presented for use in the construction of a triaxial turning dynamometer. Specifications on the design and construction of the force transducer and dynamometer have been provided. A series of calibration experiments were conducted to investigate sensitivity, linearity, hysteresis, and repeatability. In addition to modal analysis and real turning tests, the dynamic characteristics and performance of the dynamometer for the turning process were examined.

## 2. Concept Design of Turning Dynamometer

### 2.1. Cutting Force Component in Turning Process

**Figure 1:**
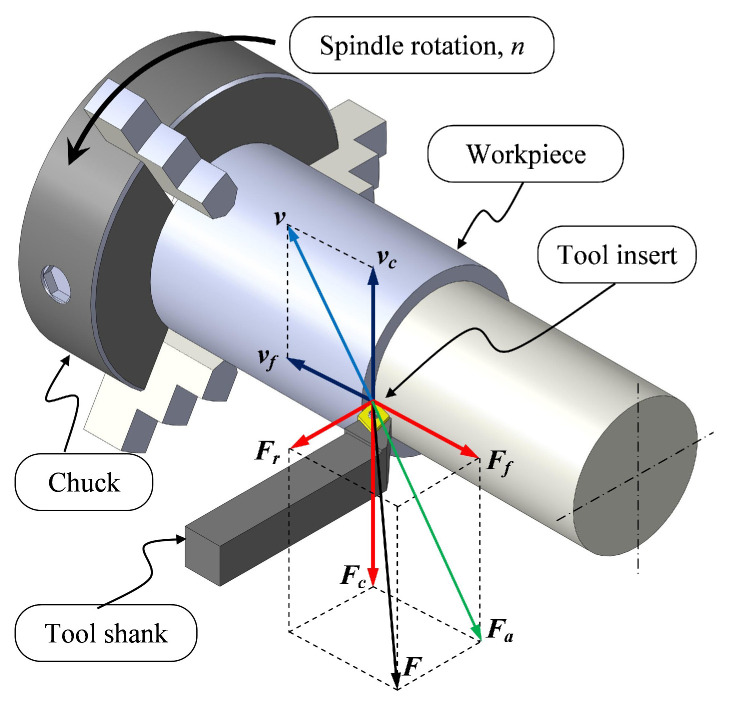
Three axes of the cutting force components in a turning process.

The turning process, which has been depicted in Figure 1, involves the workpiece material being held in a chuck in front of the spindle machine and turned at a certain rotation, n (rpm). During the process of chip removal, the tool insert will act as a barrier if it comes into contact with the material of the workpiece. This will prevent the cutting tool from penetrating the material. To compensate for this, a force *F*, referred to as the machining force or the resultant force, must be applied. This force can be analyzed by dissecting it into three distinct components, commonly referred to as the three-axis cutting force components. The active force, *F_a_*, is the result of the combination of the cutting force, *F_c_*, and the feed force, *F_f_*, in the direction of the cutting action. The passive or radial force, denoted by the symbol *F_r_*, does not contribute to the power conversion, since the tool and the workpiece do not move relative to one another in the direction of movement. The resultant force on the turning process can be expressed by the following equation:

(1) $$F=\sqrt{F_{a}^{2}+F_{r}^{2}}=\sqrt{F_{c}^{2}+F_{r}^{2}+F_{f}^{2}}$$

and:

(2) $$F_{a}=\sqrt{F_{c}^{2}+F_{f}^{2}}$$

### 2.2. Design of Force Transducer and Dynamometer

**Figure 2:**
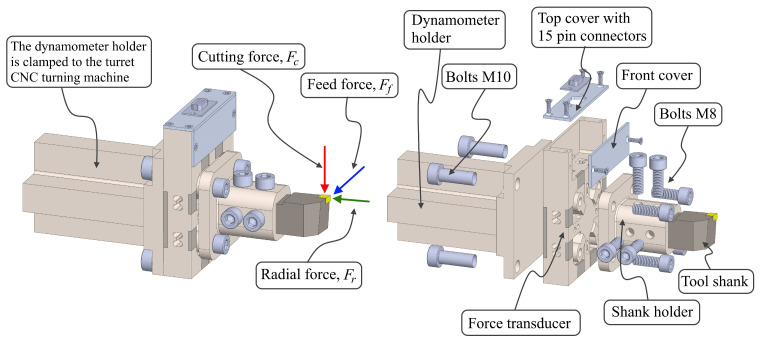
Structure of the dynamometer model and an explosion view of the primary component.

The dynamometer that had been developed, which was based on a cross-beam-type force transducer and had symmetrical grooves, was selected to serve as the elastic element for the insertion of the piezoresistive strain gauge. This transducer is positioned in between two holders, namely the dynamometer holder that is attached to the turret and the tool shank holder that is located in front of the dynamometer. Figure 2 shows the geometric design of a dynamometer and its other parts for measuring the cutting force *F_c_* in the vertical direction or the direction of the spindle rotation, the feed force *F_f_* in the feed direction, and the radial force *F_r_* perpendicular to the axis of the workpiece axis or in the direction of the tool holder.

**Figure 3:**
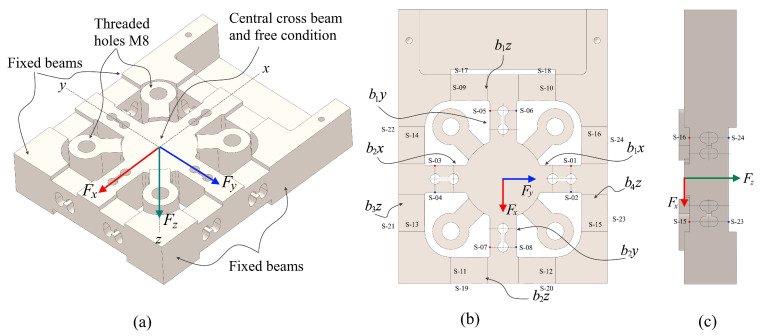
Structure of the cross-beam force transducer: (**a**) isometric view; (**b**) front view; (**c**) left side view.

This dynamometer is constructed of a number of different parts, the most important of which are the dynamometer holder, the force transducer, the shank holder, and the supporting components, which are the cover as well as the connector. The force that is applied during the machining process will be applied at the end of the tool. This force will be transmitted through the tool shank and shank holder to the force transducer surface through four threaded holes, and it will be distributed over the cross-beam with symmetrical and grooved holes, as shown in Figure 3a. This hole is there to increase the sensitivity of the sensor in the presence of a concentrated strain on the top surface of the hole. This has been designed to be symmetrical in order to adjust for the moment factor and the direction of the force at three different co-ordinates. This ensures that the deformation disturbance in one direction of the force does not impact the other sensors when they are employed simultaneously.

When the cutting forces actually act on the cutting tool tip, creating the primary cutting force *F_c_*, the feed force *F_f_*, and the radial force *F_r_*, they may correspond to forces in the co-ordinate system, namely the forces *F_x_*, *F_y_*, and *F_z_*. The three components of the cutting force will be focused on the central cross beam that is in a free condition, as shown in Figure 3a,b. Under ideal circumstances, when a force *F_x_* is applied to a force transducer, it only results in beams *b_1x_* and *b_2x_*, which generate deformation and strain. When only force *F_y_* is applied, beams *b_1y_* and *b_2y_* are deformed and strain is apparent on the surface. When the *z*-axis co-ordinates are subjected to a force *F_z_*, the beams *b_1z_*, *b_2z_*, *b_3z_*, and *b_4z_* will deform.

**Figure 4:**
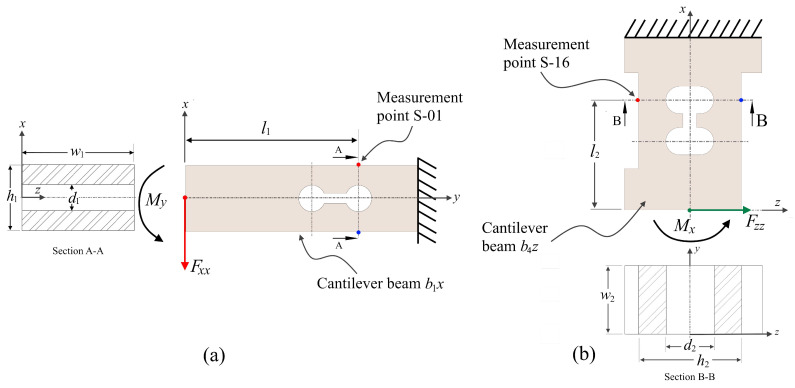
Cantilever beam model in cross-beam force transducer: (**a**) force in *x*-direction; (**b**) force in *z*-direction.

In accordance with the working mechanism of this force transducer, which has been depicted in Figure 3, a total of 24 points that have a high sensitivity and make it easy to embed strain sensors have been chosen to serve as strain measurement points. These points, which have been denoted by the numbered notation *S*-01 to *S*-24, have been labeled accordingly. For each cantilever beam, two locations are chosen as measurement points. These points are perpendicular to each other, which, in theory, provides a tension strain while the other produces a compressive strain. The locations of the measurement points (*S*-01 to *S*-08) that are on the beam to indicate the x and y forces on their corresponding cantilever beams are identical to the location of the measurement point *S*-01, which has been simplified by the model illustrated in Figure 4a. The cantilever beam measurement points (*S*-09 to *S*-24) correspond to measurement point *S*-16 in Figure 4b. Furthermore, strain measurement points are symmetrical and uniformly distributed throughout the center of the cross beam as a result of the action of the force *F_z_*. The measurement points *S*-01 and *S*-03 are symmetrical about the neutral surfaces of the cantilever beams *b_1y_* and *b_2y_*, while the measurement points *S*-05 and *S*-07 are symmetrical about the neutral surfaces of the cantilever beams *b_1x_* and *b_2x_*. The locations of the measuring points for the forces *F_x_* and *F_y_* in cantilever beams are similar to one another, as are their geometric constructions. However, with regard to the measuring point of force *F_z_*, the geometry and dimensions are the same from points *S*-09 until *S*-24. So, the measurement concept of a force transducer can be shown by looking at how two types of cantilever beams are used to measure the force (Figure 4).

In an ideal condition, the working mechanism of a three-axis force transducer only requires the cantilever beam’s *b_1x_* and *b_2x_* components to detect *F_x_*. Cantilever beams b_1y_ and *b_2y_* only need to detect F_y_, while cantilever beams *b_1z_*, *b_2z_*, *b_3z_*, and *b_4z_* only need to detect *F_z_*. The cantilever beam is composed of two symmetrical grooved holes that allow for the measurement of three-dimensional forces to be achieved. The strain value at measurement point *S*-01 is insensitive to the effects of forces *F_y_* and *F_z_* because the hole weakens the stiffness of the beam, and the strain value at measurement point *S*-05 is similarly unaffected by forces *F_x_* and *F_z_*.

When an external force is generated by a machining operation and applied in a force transducer of the cross-beam type, the force values applied to each cantilever beam are *F_xx_* = 1/2 *F_x_*, *F_yy_* = 1/2 *F_y_*, and *F_zz_* = 1/8 *F_z_*. By using the mechanical theory of the bending stress of a cantilever beam, which can be written as follows:

(3) $$\sigma_{b_{1}x}=\frac{Mh/2}{I_{b_{1}x}}$$

where *σ* = *εE*, *M* = *F_xx_l*, and *I* = *wh*^3^/12, the strain value at measurement point *S*-01 can be expressed as follows:

(4) $$\varepsilon_{b_{1}x}=\frac{F_{xx}l_{1}h_{1}/2}{I_{b_{1}x}E}=\frac{3F_{x}l_{1}h_{1}}{Ew_{1}\left(h_{1}{}^{3}-d_{1}{}^{3}\right)}$$

where *ε_b_*_1*x*_ is the strain values of measurement in beam *b*_1_*x* at point s-01 under the action of *F_x_* and *E* is the modulus of elasticity of stainless steel 316.

When force *F_z_* is applied to the transducer, beam *b_z_* will detect stress, and the value of strain on beam *b*_4*z*_ at point *S*-16 can be obtained using the following equation:

(5) $$\varepsilon_{b_{4}z}=\frac{F_{zz}l_{2}h_{2}/2}{I_{b_{4}x}E}=\frac{0.75F_{z}l_{2}h_{2}}{Ew_{2}\left(h_{2}{}^{3}-d_{2}{}^{3}\right)}$$

### 2.3. Analysis by Finite Element Method (FEM)

Using ANSYS, a numerical analysis of the cross-beam force transducer was performed to ascertain the strain distribution along the middle line of the cross-beam surface, the stiffness of the structure, and the cross-sensitivity of three axes. Several considerations, including the force being measured, rigidity, and corrosion resistance, must be made when choosing the material for the force-sensing element [1]. Stainless steel 316 L was selected because of its high rigidity and good corrosion resistance. It has a density of 8000 kg/m^3^, Young’s modulus of 193 GPa, a Poisson ratio of 0.27, and a yield strength of 520 MPa.

This study requires the force transducer to be securely fastened within the dynamometer holder as a boundary condition. The shank end tip of a tool holder was subjected to a load of 2000 N, evenly distributed. The results of this analysis were supposed to reveal the strain distribution over the through-hole surface of the beam at strain sensor locations and the hub center displacement resulting from the applied force. 

Wheatstone bridges are utilized in the construction of the three-axis force transducer’s individual channels. It is feasible to estimate the rated strain of each axis component sensor under each rated force by making use of the strain values that are highest along the midline of the possible placement of piezoresistive strain gauges on each channel, as illustrated in Figure 3.

(6) $$\varepsilon_{F_{x}}=\frac{1}{4}(\varepsilon_{S\text{-}01}-\varepsilon_{S\text{-}02}+\varepsilon_{S\text{-}03}-\varepsilon_{S\text{-}04})$$

(7) $$\varepsilon_{F_{y}}=\frac{1}{4}(\varepsilon_{S\text{-}05}-\varepsilon_{S\text{-}06}+\varepsilon_{S\text{-}07}-\varepsilon_{S\text{-}08})$$

(8) $$\varepsilon_{F_{z}}=\frac{1}{4}\left[\begin{array}{l} (\varepsilon_{S\text{-}09}+\varepsilon_{S\text{-}10}+\varepsilon_{S\text{-}11}+\varepsilon_{S\text{-}12})-(\varepsilon_{S\text{-}17}+\varepsilon_{S\text{-}18}+\varepsilon_{S\text{-}19}+\varepsilon_{S\text{-}20})+\\ \left(\varepsilon_{S\text{-}13}+\varepsilon_{S\text{-}14}+\varepsilon_{S\text{-}15}+\varepsilon_{S\text{-}16})-(\varepsilon_{S\text{-}21}+\varepsilon_{S\text{-}22}+\varepsilon_{S\text{-}23}+\varepsilon_{S\text{-}24}\right) \end{array}\right]$$

where *εF_x_*, *εF_y_*, and *εF_z_* are the total strains from the Wheatstone bridge for each direction of force and *S_i_* is the strain measured on the cross-beam surface in a certain position (*i* = 1 to 16). 

**Figure 5:**
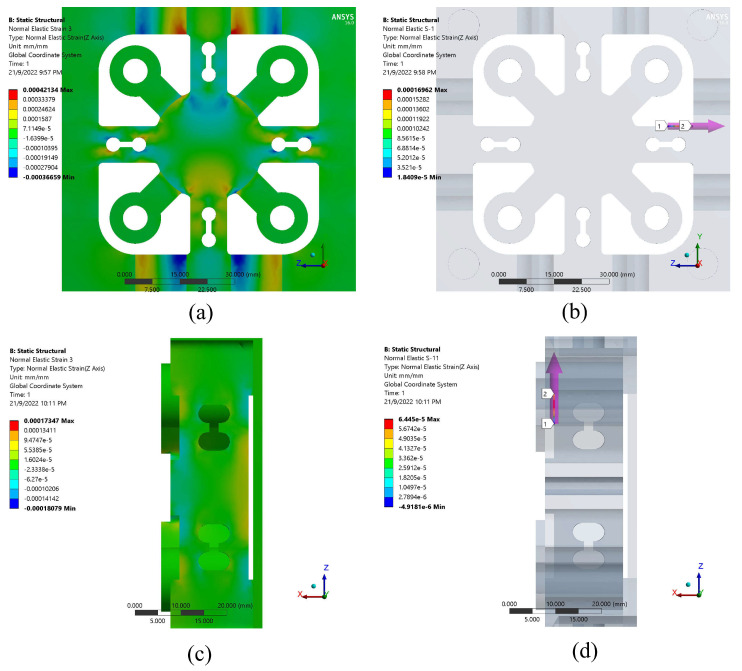
Strain distribution on cross-beam surfaces while under: (**a**,**b**) *F_x_* force; (**c**,**d**) *F_z_* force.

Figure 5a,b show the results of the strain distribution on the cross-beam surface areas brought on by the *x*-axis force. The areas around the through-grooved hole surfaces showed the highest levels of strain. Due to its symmetrical design, the strain induced by a force will have the same magnitude whether measured along the *x-* or *y*-axis. Figure 5c,d display the strain distribution throughout the cross-beam surface due to a force applied along the *z*-axis. Maximum strain is seen on the surface that is closest to the fixed zone. 

**Figure 6:**
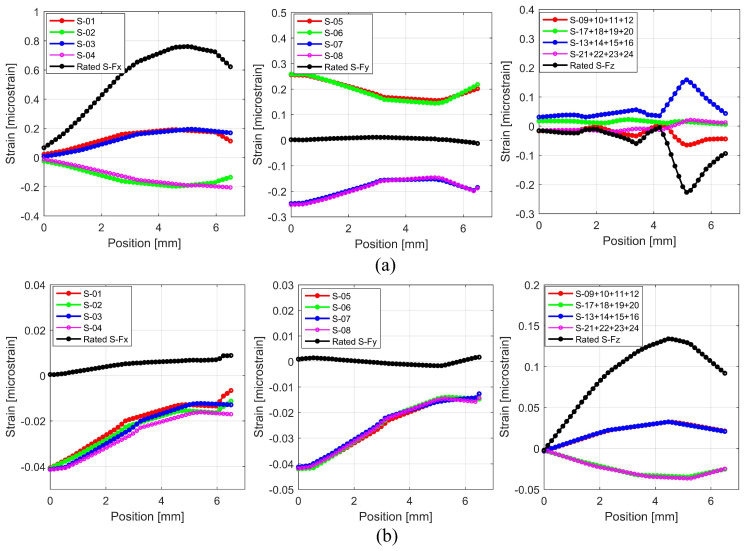
Normal strain along the midline of cross-beam surfaces: (**a**) when *F_x_* force is applied; (**b**) when *F_z_* force is applied.

A plot of the normal strain along the cross-beam midlines as it appears on their surfaces is shown in Figure 6. Because of the symmetrical nature of the construction, the normal strain was distributed in a manner that was similar along both the x and y axes. Due to the force *F_x_* operating on the force transducer, the maximum strain on the *x*-axis was 0.75 microstrain at locations 4 to 6 mm in Figure 6a. In comparison, the strains on the *y* and *z* axes were 0.005 and −0.2 microstrain at the same position. This suggests that the interference strain that was caused by a force *F_x_* on the *y* and *z* channels was relatively small or even nonexistent. The highest amount of strain that was measured as a result of a radial force, denoted by *F_z_*, was 0.13 microstrain, whereas the amount of strain that was measured in the x and y channels was around 0.004 and −0.0002 microstrain, respectively.

## 3. Fabrication of the Tri-Axial Turning Dynamometer

### 3.1. Piezoresistive Strain Gauge Arrangement in Cross-Beam Force Transducer

By attaching a piezoresistive strain gauge to the cross-beam force transducer’s elastic surface, the resistance of the strain sensor can be made to change in response to the applied force. A Wheatstone bridge circuit can be used to transform the strain into an electrical signal. The proper placement of the strain sensor, as discussed in the FEM results section and depicted in Figure 3 above, has been demonstrated. An integral part of this work is the strain sensor layout on the transducer structure, which must be completed before the turning dynamometer can be installed.

**Figure 7:**
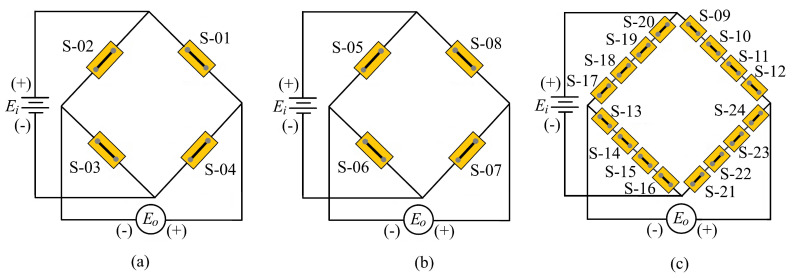
Wheatstone bridge circuits for detection of forces in cross-beam force transducer: (**a**) *F_x_*; (**b**) *F_y_*; (**c**) *F_z_*.

Figure 7a depicts the strain sensor configuration for sensing the force in the *x*-direction or the main cutting force Fc. Strain sensors *S*-01 and *S*-03 are subjected to tension, whereas strain sensors *S*-02 and *S*-04 are subjected to compression. The *y*-direction force or feed force, *F_f_*, is detected by strain sensors *S*-06 and *S*-08, which are subjected to compressive stress, as indicated in Figure 7b, whereas strain sensors *S*-05 and *S*-07 are exposed to tensile stress. As seen in Figure 7c, the *z*-direction force or radial force, *F_r_*, is detected by strain sensors *S*-09, *S*-10, *S*-11, and *S*-12; and *S*-13, *S*-14, *S*-15, and *S*-16 are exposed to a tensile stress, whereas strain sensors *S*-17, *S*-18, *S*-19, and *S*-20 and *S*-21, *S*-22, *S*-23, and *S*-24 are subjected to compressive stress. All strain sensors used in this work were linear semiconductor or piezoresistive strain gauges SSC-350-B-F5 (UTOP) with the nominal resistance of 350 Ω. The gauge factor (GF) of this sensor was 150, the length of the gauge was 6 mm, and the width of the gauge was 3.5 mm. The actual strain could be obtained from the following equation [1]:

(9) $$\frac{\Delta L}{L}=\varepsilon =\frac{\Delta R/R}{GF}$$

where *ε* is the strain detected by the strain gauge, *R* is the initial resistance of the sensor, Δ*R* is the differential resistance, *L* is the original length, and Δ*L* is gauge elongation due to stress. 

### 3.2. Constraction of Dynamometer and Data Acqusition System

The structure of the tri-axial dynamometer consisted of the dynamometer holder, the tool shank holder, the cross-beam force transducer, and piezoresistive strain gauges that were attached as sensor elements and supporting components, as well as the electrical connections that were necessary to create Wheatstone bridge circuits. The material for the force transducer is stainless steel 316L. Three channels of cutting force signals were sent to the data collection system of the NI-9237 through a multi-conductor wire after being sent through the connection on the dynamometer unit. The NI-9237 had a maximum sampling rate of 50 kHz and a 24-bit A/D converter in its data acquisition system. However, in this study, the sampling rate used is only 5 kHz because the cutting dynamics in the turning process is still two times below that frequency range. After that, the analog data included in the signals was transformed into digital data, and the Signal-Express program was used to record and display the results. 

## 4. Testing of Tri-Axial Turning Dynamometer

### 4.1. Static Calibration Test 

**Figure 8:**
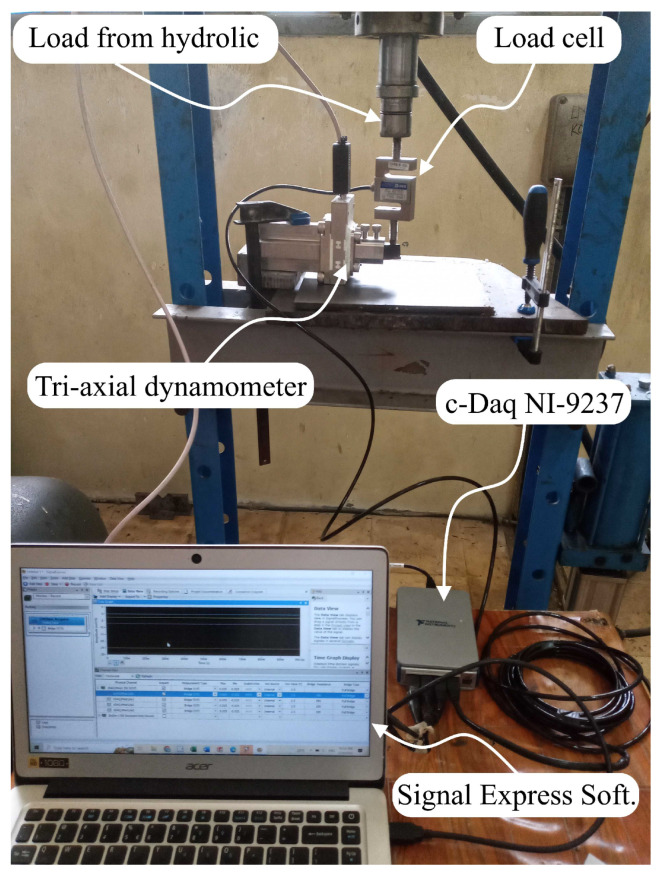
Dynamometer setup in static calibration test.

In order to test the performance of the dynamometer after its design and completion of construction, static calibration was carried out with the use of a hydraulic press that was fitted with a standard load cell (Zemic-H3-C3). The configuration setup of the static calibration test is shown in Figure 8. In this experiment, increasing loads of 0 to 2000 N were applied in increments of 50 N to each of the three different orientations (*x*, *y*, and *z*).

In order to establish a correlation between the mean values of the outputs and the measured cutting forces, calibration graphs were constructed. When measurements were taken for one channel, data from all of the other channels were also obtained at the same time. As a result, research was conducted to investigate how the effects of each loading varied according to orientations other than its own. In Figure 8, the calibration graphs for the forces along the *x*-direction (*F_x_*), the *y*-direction (*F_y_*), and the *z*-direction (*F_z_*), as well as their interactions, have been displayed.

The measurements were carried out five times, and the findings of the average measurements were plotted on a graph. This was conducted to confirm that the results were reliable. Because the cross-beam force transducer could be thought of as a linear system, the following relationship can be shown:

(10) S=[C]F

(11) $$\left[\begin{array}{c} S_{F_{x}}\\ S_{F_{y}}\\ S_{F_{z}} \end{array}\right]=\left[\begin{array}{ccc} C_{11} & C_{12} & C_{13}\\ C_{21} & C_{22} & C_{23}\\ C_{31} & C_{32} & C_{33} \end{array}\right]\left[\begin{array}{c} F_{x}\\ F_{y}\\ F_{z} \end{array}\right]$$

where *F* is a vector of the force value [*Fx Fy Fz*] applied by the input component, *C* is a matrix element or calibration matrix, and *S* is a vector of the voltage generated by the dynamometer. The static calibration matrix can be obtained in the following manner, in accordance with the findings of the experiments:

$$\left[\begin{array}{ccc} 32.799 & 0.532 & -1.684\\ 0.185 & 23.269 & 1.084\\ 0.401 & -0.125 & 7.741 \end{array}\right]$$

The calibration curve and matrix made it plainly evident that the sensitivities of the tri-axial turning dynamometer were acquired at around 32.799 µV/N, 23.269 µV/N, and 7.741 µV/N. Table 1 displays the cross-talk error for each of the *Fx*, *Fy*, and *Fz* directions separately. The largest error for the *Fz* component was around 5.17% in the *Fx* direction, but the error for the other components ranged between 0.79 and 1.62% in any direction. This is evidence of a successful design with low levels of cross-sensitivity and indicates that the coupling intensity is low. Cross-sensitivity is a complex phenomenon; yet, it is extremely difficult to collect precise conclusions regarding it due to the large number of external factors that are involved [17].

**Figure 9:**
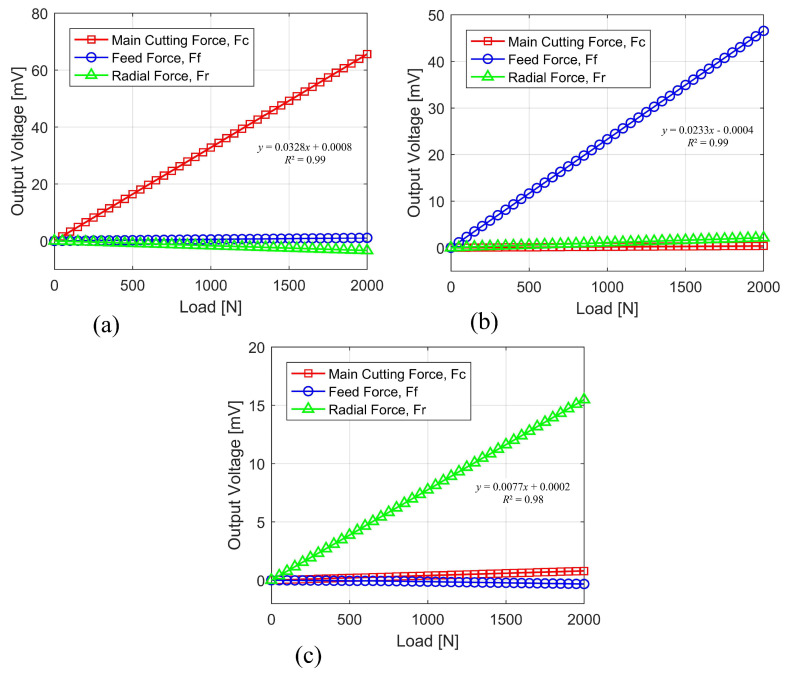
Dynamometer calibration curves: (**a**) *F_x_* direction; (**b**) *F_y_* direction; (**c**) *F_z_* direction.

To further evaluate its static features, the constructed tri-axial turning dynamometer’s linearity, hysteresis, and repeatability were studied for each of the three directions of forces. The linearity of the bridge’s output reading can be gauged from Figure 9, which shows the output to force conversion for each component. Loads of 500 N, 1000 N, and 1500 N were applied to each part to test the linearity of the instrument and see if it could measure loads outside of its normal range of operation. Gradually increasing the loading from 0 to 2000 N and, likewise, for each component, the hysteresis test validated the difference between reading the increasing load and reading the decreasing load on the dynamometer. The dynamometer’s reliability was also assessed by testing its capacity to produce the same results again. Five sets of loading at 10 N, 50 N, and 100 N were used, for a total of 15 measurements across all components. Two parameters were selected to demonstrate the repeatability error: the mean and the standard deviation. The linearity, hysteresis, and repeatability of the applied forces have been shown in Table 2. The dynamometer’s maximum error rates for linearity and hysteresis were clearly below 1%, but repeatability error also did not exceed 3%.

### 4.2. Dynamic Test of Dynamometer

When a turning dynamometer is used in the machining process, the cutting force does not remain static; as a result, it is necessary to take into account how the system responds when subjected to dynamic excitation. The dynamic response is affected by the natural frequency of the structure of the dynamometer. During the machining process, the natural frequency should be higher than the excitation vibration frequency. This will guarantee that the recorded cutting force signal will not be impacted by the dynamic reaction caused by the dynamometer’s construction. The experimental modal analysis is used to obtain the frequency response function, which is then used to find the dynamometer’s natural frequency [1].

**Figure 10:**
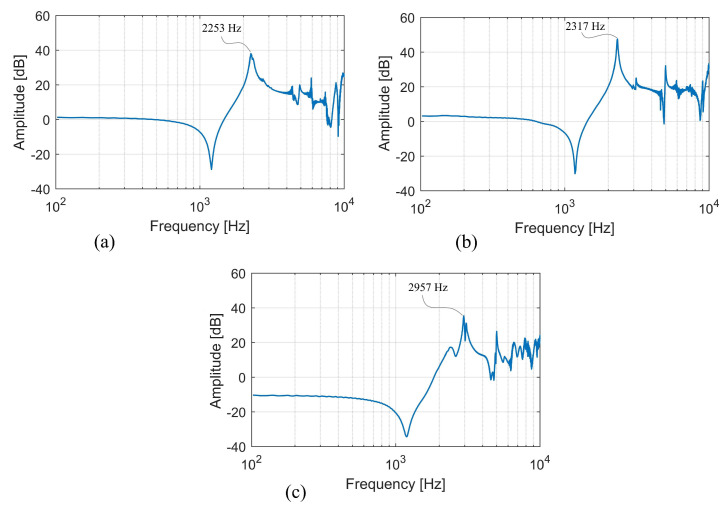
Frequency response of the dynamometer: (**a**) x-direction; (**b**) y-direction; (**c**) z-direction.

A Piezotronics impulse force hammer (086C03) with a vinyl tip sensitivity of 2.32 mV/N was used to excite the dynamometer in three different directions (the *x*-axis, the *y*-axis, and the *z*-axis), and a Piezotronics accelerometer (352C33) with a sensitivity of 10.35 mV/m/s^2^ was mounted onto the dynamometer component. A sampling rate of 50 kHz was used in the LabVIEW program in order to acquire the signal, which was then collected by the data acquisition device (NI-9250). After that, a modal analysis was carried out in order to determine the frequency response function of the dynamometer in three co-ordinate directions in accordance with the three-axis force components. The graphs of the natural frequencies acquired by dynamic testing and modal analysis are shown in Figure 10. The turning dynamometer’s construction had natural frequencies of 2253 Hz, 2317 Hz, and 2957 Hz, respectively, when measured along the axes *x*, *y*, and *z*. These results revealed that the specified dynamometer construction was excellent for both conventional turning and high-speed turning.

### 4.3. Performance Test of Dynamometer in Turning Operation

**Figure 11:**
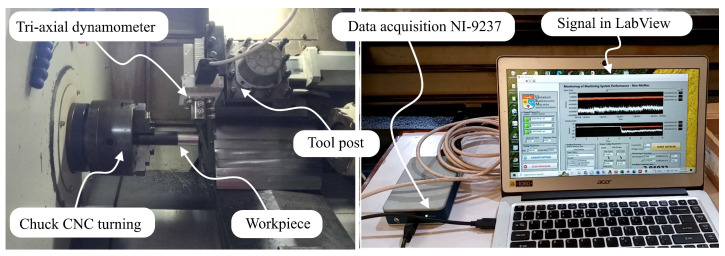
Machining setup for performance test of developed dynamometer.

Testing the capability of the developed tri-axial dynamometer to measure cutting forces during real machining operations is an important stage in the evaluation process. The experiment was carried out on a CNC turning machine (Focus NX-L300) in a dry environment using straight turning with three distinct depths of cut, particularly 0.25 mm, 0.50 mm, and 0.75 mm, respectively. Carbide cutting tool inserts (DCMT 070204) were used during the tests, which were carried out on rods of AISI 1045 carbon steel, 32 mm in diameter. Both the feed rate and the spindle speed were held constant at 0.1 mm/rev and 512 rpm, respectively. The actual setup for the machining test can be seen in Figure 11.

**Figure 12:**
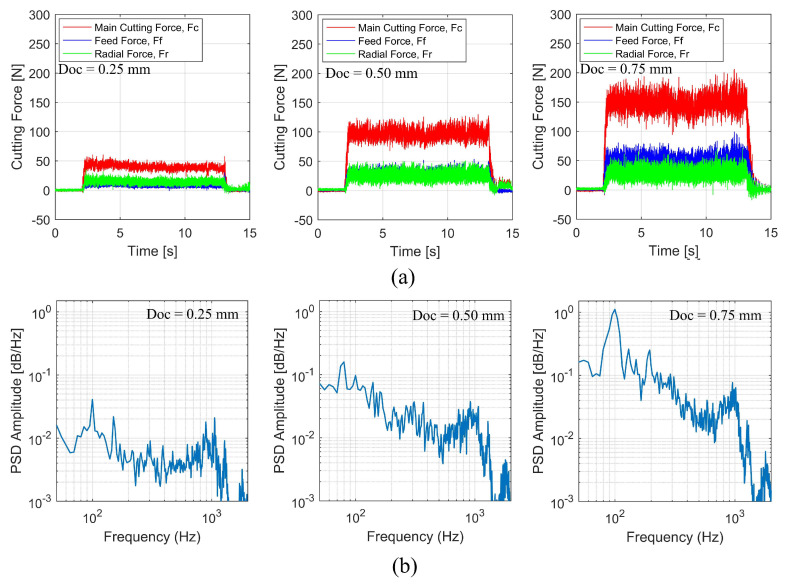
Cutting forces in turning operation under three different depths of cut: (**a**) time domain; (**b**) frequency domain.

In Figure 12a, the tri-axial cutting forces that were recorded in straight turning have been depicted under three distinct conditions involving the depth of cut. The change in cutting force is very significant when the tool engages the workpiece and the signal reading continues straight during machining until the tool is out of the cut zone. It can be seen that the main cutting force increases as the depth of cut increases and that they maintain a stable relationship with each other. This shows that the dynamometer can accurately show how the cutting force changes as the cutting parameters change.

Variations in the depth of cut lead to dynamic shifts, which also bring about significant changes. The time domain plot makes it abundantly evident that there is an increase in signal fluctuation whenever there is a change. This is also apparent when the cutting is maintained during a long operation. It is also clearly evident that an increase in the depth of cut would result in an increase in the power spectral density, as seen in Figure 12b. These findings suggest that the readings generated by the developed dynamometer are able to reliably detect shifts in parameter conditions across all three of the signal channels that are being evaluated.

## 5. Conclusions

In this study, a novel cross-beam force transducer was designed and manufactured for the development of a tri-axial turning dynamometer for measuring the three cutting force components. Numerous highly sensitive piezoresistive strain gauges were utilized in this dynamometer. To efficiently create a force transducer structure, strains with Wheatstone bridge circuits were investigated using finite element analyses in order to establish the position of the piezoresistive strain gauge. This dynamometer was designed to measure up to 2000 N of main cutting force, feed force, and radial force. The static, dynamic, and real machining tests were performed in order to evaluate the dynamometer’s performance. The developed dynamometer had good linearity, repeatability, and hysteresis, as well as the capacity to reliably detect main cutting force, feed force, and radial force sensitivities of 32.799 µV/N, 23.269 µV/N, and 7.741 µV/N, respectively, with cross-sensitivity errors of less than 3%. Dynamic analysis and testing revealed that the stationary dynamometer’s natural frequencies were around 2253 Hz, 2317 Hz, and 2957 Hz in the *x*, *y*, and *z* directions, respectively. This indicated that the structure’s stiffness and dynamic range were suitable for both standard and high-speed machining operations. Based on the results of real machining experiments, the designed tri-axial turning dynamometer is an effective method to measure the dynamic cutting force in turning operations.

## Figures and Tables

**Table 1:** Cross-talk error of the dynamometer based on static calibration test.

Force Direction	Cross-Talk Error (%)		
	*F_x_*	*F_y_*	*F_z_*
*F_x_*	-	1.62	5.13
*F_y_*	0.79	-	4.66
*F_z_*	5.17	1.62	-

**Table 2:** Linearity, hysteresis, and repeatability errors for each axis of the dynamometer.

Force Direction	Avg. of Linearity Error (%)	Hysteresis Error (%)	Repeatability Error (%)		
			10 N	50 N	100 N
*F_x_*	0.02	0.36	0.27	0.38	0.86
*F_y_*	0.06	0.62	0.77	1.63	1.94
*F_z_*	0.13	0.46	0.82	2.79	2.91

## Data Availability

The data presented in this study are available on request from the corresponding author.
